# Systemic Lupus Erythematosus and Hereditary Coproporphyria: Two Different Entities Diagnosed by WES in the Same Patient

**DOI:** 10.1155/2022/9096999

**Published:** 2022-05-28

**Authors:** Anlei Liu, Lingli Zhou, Huadong Zhu, Yi Li, Jing Yang

**Affiliations:** Emergency Department, State Key Laboratory of Complex Severe and Rare Diseases, Peking Union Medical College Hospital, Chinese Academy of Medical Science and Peking Union Medical College, Beijing, China

## Abstract

**Background:**

Hereditary coproporphyria (HCP) is a rare autosomal dominant disorder caused by a partial deficiency of coproporphyrinogen III oxidase (CPOX), and systemic lupus erythematosus (SLE) is an autoimmune disease with a strong genetic predisposition. *SLC7A7* (solute carrier family 7 member 7) may be associated with monogenic lupus disease; however, only 2 cases of concomitant HCP and SLE have been reported.

**Methods:**

We report a 30-year-old woman with a six-year history of SLE presenting with abdominal pain, vomiting, dysuria, tachycardia, and hyponatremia. Whole exome sequencing (WES) and Sanger sequencing were carried out for the proband and members of her pedigree to detect the genetic background. The Gene Expression Omnibus (GEO) database was used to search the related gene expression profiles. Differentially expressed genes (DEGs) were identified using GEO2R.

**Result:**

A novel heterozygous splicing mutation of *CPOX* (NM_000097): c.700+2 T > C (intron 2) was detected by WES in the proband, and it was considered likely pathogenic (PSV1+PM2). Sanger sequencing verified the heterozygous mutation of *CPOX* in the proband, although it was not detected in her father. WES also identified 62 other gene variants, especially two heterozygous variants in *SLC7A7* (NM_001126106): c.250G > A (p. V84I) and c.625+1G > A (splicing). DEGs were detected from GSE51997, and the expression of *CPOX* was downregulated in SLE patients compared with normal controls (adj. *P* = 0.0071, logFC = −1.0975).

**Conclusion:**

This study presents the first reported case of SLE coexisting with HCP in China; moreover, a novel splicing mutation of *CPOX*, i.e., c.700+2 T > C (intron 2), and two heterozygous mutations of *SLC7A7* were reported. The simultaneous mutations of *CPOX* and *SLC7A7* may explain the etiopathogenetic connections of HCP and SLE.

## 1. Introduction

Hereditary coproporphyria (HCP) is a kind of acute hepatic porphyria (AHP) and a rare autosomal dominant disorder caused by genetic mutation in coproporphyrinogen III oxidase (CPOX) [1]. Patients with HCP can present with serious abdominal, psychiatric, neurologic, cardiovascular, or cutaneous symptoms [1]. Systemic lupus erythematosus (SLE) is an autoimmune disorder in which autoantibodies that damage multiple organs are produced [2]. Genetic predisposition plays an important role in SLE etiology [3]. Over thirty genes were reported to be related to monogenic lupus disease or lupus-like diseases, including *SLC7A7* (solute carrier family 7 member 7 gene) [4–6]. The coexistence of AHP and SLE is extremely rare [7–10]. We reported a difficult diagnosis of HCP in a patient with a six-year history of SLE, in which HCP was caused by a novel *CPOX* variant and SLE might have been related to the *SLC7A7* variant background.

## 2. Materials and Methods

### 2.1. Case Report

A 30-year-old woman with SLE was admitted to the emergency department because of abdominal pain.

On September 15, 2020, she developed a sore throat and cough and was subsequently treated with roxithromycin, ambroxol, and Honghua (a kind of Chinese traditional medicine). On September 17, acute and severe abdominal pain and cramping developed, with concomitant left low back pain, vomiting, and dysuria but no venting or defecation. She presented to a local hospital, where a clinical diagnosis of intestinal obstruction was made and fasting, gastric intubation, and support treatments were prescribed. The patient felt no release of abdominal pain and was transferred to the emergency department of this hospital on September 22. She had a background of pulmonary tuberculosis twenty years ago and was treated with isoniazid and rifampicin for two years. The patient was diagnosed with immune thrombocytopenia (ITP) in 2002, she suffered from frequent hemorrhage for ten years and finally underwent splenectomy in 2012, and she presented with an itchy rash on her face and limbs after exposure to sunlight in 2014. Autoantibody testing showed that antinuclear antibody (ANA) was elevated (1 : 640); Sm, SSA, RO 52, and Scl 70 antibodies were positive, while double-stranded DNS (ds-DNA) antibodies were negative. No other organ involvement was noticed. She was diagnosed with SLE and administered hydroxychloroquine (HCQ, 0.4 g/d) and prednisone (7.5 mg/d). Her last menstruation began on September 20, exactly the third day after abdominal pain developed. She denied any alcohol, tobacco, or drug abuse. Her family history was significant for lung cancer (mother died of lung cancer).

On admission, oral examination revealed oral mucosal dryness and bleeding and several lower lip mucosa ulcers. The abdomen was soft, with decreased bowel sounds, and tenderness was noticed around the bellybutton. Laboratory studies revealed hyponatremia (128 mmol/L) and increased C-reactive protein (14.07 mg/L). Liver enzymes, bilirubin, albumin, creatinine, uric acid, glucose, cholesterol, serum amylase and lipase, thyroid tests, erythrocyte sedimentation rate, and complement (C3 and C4) tests were normal. Antinuclear antibody (ANA) levels were elevated (1 : 160), SSA 60 and RO 52 antibodies were positive, and double-stranded DNA (ds-DNA) and other nuclear antigen antibodies were negative. Electrocardiogram showed sinus tachycardia. Abdominal and pelvic CT scans revealed a soft tissue signal behind the uterus and a suspicious small stone at the pelvic part of the ureter. Tramadol and chlorpromazine were administered to control the abdominal and back pain. Therapy was initiated with ertapenem (1 g/d) and methylprednisolone (0.5 mg/kg body weight) because of the infection, and the medication prescribed for SLE was suspended because of the sudden onset of symptoms. Oral laxative and intravenous infusion of 10% glucose and saline were administered due to suspicious intestinal obstruction.

**Figure 1:**
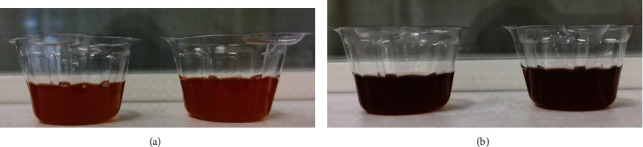
Urine appearance. (a) Reddish urine of the patient suffering acute porphyria attack. (b) Dark brown urine after one-hour exposure to sunlight.

The patient presented with worsening weakness and dysuria after 24 hours of admission. Reddish urine was noticed after indwelling a urinary catheter. This finding raised the suspicion of acute porphyria. The urine turned a dark brown color after one hour of exposure to sunlight (Figure 1). The urobilinogen level was 33 *μ*mol/L (reference range, 3 to 16 *μ*mol/L), and the uroporphyrinogen (UPG) and porphobilinogen (PBG) tests were positive. Free erythrocyte protoporphyrin (FEP) was 5.3 *μ*g/gHb (reference range, 0 to 4.7 *μ*g/gHb). Brain magnetic resonance imaging did not reveal any pathologic features.

Based on these findings, we made a tentative diagnosis of acute porphyria. Ertapenem and methylprednisolone were stopped as the neutrophil levels returned to normal. Sufficient glucose support (250-300 g/d), restriction of liquids (<2500 mL/d), and moderate sodium supplements were administered. Morphine (5 mg) was given to relieve pain when necessary. Hydroxychloroquine (0.4 g/d) and prednisone (7.5 mg/d) were restarted for SLE. Within a four-month follow-up, no abdominal symptoms were observed.

Based on these findings, we decided to examine the genetic causes of the disease in her family.

### 2.2. Molecular Analysis

Total DNA was extracted from peripheral blood leukocytes of the patient and her father using standard methods. Whole exome sequencing (WES) was performed using Gencap™ Human whole Exon Probe V4.0 (Mygenostics, Beijing, China), and exome libraries were sequenced using the GenCap™ Platform (Mygenostics, Beijing, China). Sanger sequencing was used to confirm the variants.

### 2.3. Data Analysis and Variant Interpretation

Sequence reads were aligned to the February 2009 human reference sequence (GRCh37/hg19) assembly. Protein function was predicted by the rare exome variant ensemble learner (REVEL). Pathogenicity analysis of the mutation was performed in the Clinically Relevant Variants (ClinVar) database. Variants were numbered according to the Human Genome Variation Society (HGVS) recommendations for variant nomenclature (https://www.HGVS.org/varnomen).

### 2.4. Microarray Data Resource

A gene expression profile dataset (GSE51997) based on data obtained from the Gene Expression Omnibus (GEO) database (http://www.ncbi.nlm.nih.gov/geo/) was produced on the GPL570 Affymetrix Human Genome U133 Plus 2.0 Array. According to the annotation information in the platform, the probes were alternated with corresponding gene symbols. GSE51997 contains eight CD16^−^ inflammatory monocytes from normal specimens and four CD16^−^ inflammatory monocytes from active SLE specimens.

### 2.5. Identification of DEGs

The online tool GEO2R (http://www.ncbi.nlm.nih.gov/geo/geo2r) was applied to screen differentially expressed genes (DEGs). GEO2R is an intelligent web device that empowers clients to compare at least two datasets in a GEO series to distinguish DEGs. The adjusted *P* values (adj. *P*) and Benjamini and Hochberg false discovery rates were generated to ensure consistency among methods used to measure large quantities of data and control the false-positive rate.

## 3. Results

### 3.1. Molecular Analysis

**Figure 2:**
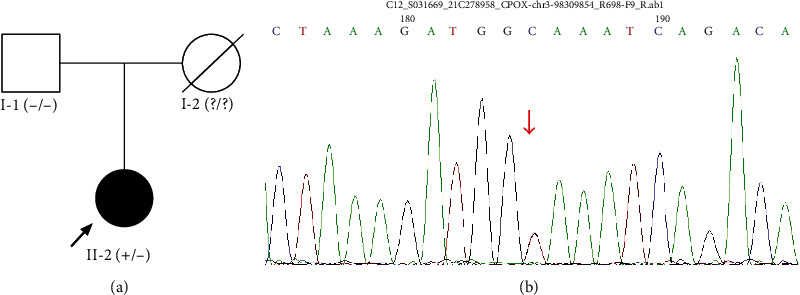
Novel *CPOX* heterozygous mutation was identified. (a) Pedigree with *CPOX* mutation (arrow indicated proband). (b) Novel *CPOX* splicing mutation, c.700+2 T > C (intron 2), was identified in the proband.

A novel CPOX gene (NM_000097) heterozygous mutation, c.700+2 T > C (intron 2), was detected by WES and confirmed by Sanger sequencing in the proband, which led to a splicing mutation (Figure 2). The diagnosis of HCP was determined in the proband. This variant is absent in public population genomic databases, and the protein function of this variant was predicted to be unknown. According to American College of Medical Genetics (ACMG) variant interpretation guidelines, the variant is preliminarily considered likely pathogenic (PSV1+PM2).

Sanger sequencing was used to detect the *CPOX* fragment of the proband's family members. The same mutation was not detected in her father. No genetic data of the patient's mother were obtained because she died from lung cancer ten years ago. No similar rash, abdominal pain, or elevated liver enzymes were noticed in her mother.

WES also identified 62 other gene variants, from which only *SLC7A7* has been reported to be associated with monogenic lupus disease. WES showed compound heterozygous variants in *SLC7A7* (NM_001126106): c.250G > A (p. V84I) in exon 3 and c.625+1G > A (splicing) in intron 4.

### 3.2. Data Quality Assessment and Identification of DEGs

**Figure 3:**
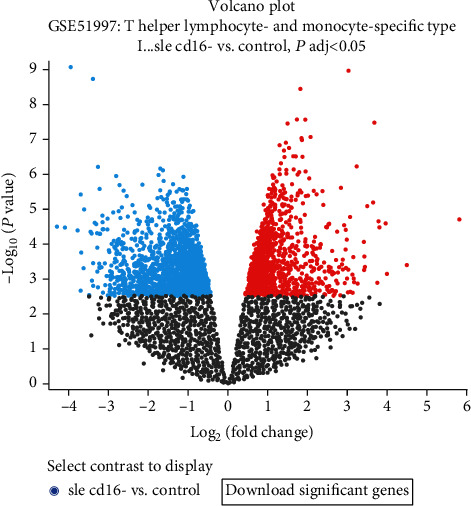
Volcano plot of DEGs between SLE patients and healthy controls in the GSE51997 dataset.

Heatmap and unsupervised clustering analyses were performed to reveal the distribution of gene expression data in GSE51997 (Figure 3). DEGs were detected from GSE51997 by GEO2R (adj. *P* < 0.01, |log*FC*| > 1). The expression of *CPOX* was downregulated in SLE patients compared with normal controls (adj. *P* = 0.0071, logFC = −1.0975).

## 4. Discussion

Acute hepatic porphyria (AHP) is differentiated into four types: acute intermittent porphyria (AIP), variegate porphyria (VP), hereditary coproporphyria (HCP), and aminolaevulinic acid dehydratase-deficient porphyria (ALADP). The main data of the 15 reported cases showing the coexistence of AHP and SLE, including the present case, are summarized in Table 1. AIP, the most common type of AHP, was reported to coexist with SLE in twelve cases [7, 8, 11–15]. Genetic defects, metabolic defects, and acquired autoimmune phenomena have been proposed to explain the coexistence of these two diseases [7–10]. Latent AIP might become clinically active by the pathological process of SLE or SLE treatment [8]. Rifampicin is porphyrinogenic, and HCQ is possibly porphyrinogenic [7, 16]. An overview of the patient's medication showed that the above drugs were not likely to represent direct triggers responsible for this attack. Meanwhile, there was no sign of SLE activity. This attack might have been triggered by upper respiratory tract infection and menstruation.

As porphyria can mimic symptoms and signs of other common disorders, a high frequency of misdiagnosis or delayed diagnosis is observed [1, 9, 17]. However, porphyrias can be easily defined and diagnosed according to the overproduction of porphyrin precursors [1]. Several puzzling points hindered early diagnosis of this case. The patient had a medical history of SLE and splenectomy and suffered severe abdominal pain immediately after an infection. In addition, an abnormal soft tissue signal behind the uterus and several small ureteral calculi were shown on CT. SLE-related intestinal pseudoobstruction and postoperative adhesive intestinal obstruction were considered first, and pelvic neoplasm and ureteral calculi were also taken into account. The keys to the diagnosis of porphyria were reddish dark urine, positive UPG and PBG, and increased FEP. The symptoms developed in this patient, including abdominal pain, vomiting, dysuria, tachycardia, hypertension, and hyponatremia, could all be explained by an attack of AHP. Finally, the diagnosis of HCP was confirmed by a *CPOX* mutation identified by WES.

HCP is the rarest of the autosomal dominant AHPs and is characterized by a deficiency of CPOX, the sixth enzyme in the heme biosynthetic pathway. HCP has over 50 reported mutations that lead to variations in penetrance and phenotype [1]. We detected a novel *CPOX* heterozygous mutation in the proband: c.700+2 T > C (intron 2). This variant caused a splicing mutation, and a part of intron 2 could be expressed following exon 2, which may result in a loss of gene function. The patient presented with serious abdominal, psychiatric, and cardiovascular symptoms but no cutaneous symptoms. Her father did not carry the same mutation, and the genic background of her mother was lacking. Although HCP is inherited in an autosomal dominant manner, the proband's mother did not have a history of symptoms similar to those of HCP. We could not determine whether the mutation originated in the proband or was inherited from her mother. Acute porphyria has low penetrance, and approximately 90% of affected individuals never experience an acute attack [18]. The allele frequency distribution of missense, nonsense, and splice-site variants is also different in various ethnic and demographic populations for porphyrias [19]. Lambie et al. [17] reported a novel missense mutation of HCP with 100% penetrance in a family. Fukui et al. [20] reported a case of HCP with a homozygote missense mutation presenting with a mild clinical phenotype. The novel *CPOX* variant in our patient may be associated with an HCP phenotype that does not have cutaneous involvement.

To the best of our knowledge, only two cases of HCP coexisting with SLE have been reported in the literature [9, 10]. Alioua et al. [10] reported on the coexistence of SLE and severe hepatic porphyria, and HCP was considered the most likely diagnosis. As Korkmaz [9] described, HCP was diagnosed in a patient with SLE and ankylosing spondylitis after 5 years of follow-up; therefore, they suggested that the genetic background of the autoimmune response might play a role in patients with porphyria [9]. In the reported cases, the diagnosis of HCP was made by metabolite testing, and genetic testing was lacking. In the present case, WES was first used to detect the genetic background underlying the coexistence of HCP and SLE.

SLE shows a strong genetic predisposition, and more than 100 susceptibility genes have been identified [21]. Moreover, novel genetic loci have been revealed recently [21–24]. *SLC7A7* maps to chromosome 14q11.2 (11) and encodes amino acid transporter 1 (y+LAT1). Lysinuric protein intolerance (LPI), a rare autosomal recessive disease, is caused by biallelic pathogenic variants in *SLC7A7*. Autoimmunity and immunological abnormalities have been observed in patients with LPI, including SLE [25–27]. A significant number of genes associated with autoinflammation and autoimmunity have been implicated in monogenic lupus, including *SLC7A7* [4–6]. The compound heterozygous variants in *SLC7A7*, namely, c.475C > T (p. Arg159Cys) and c.1001 T > G (p. Leu334Arg), were detected in a patient who clinically presented with immune dysregulation in the setting of early onset SLE [28]. Li et al. [27] reported five mutations of *SLC7A7*: c.625+1G > A, c.235G > A, c.1085 T > C, c.1387delG, and c.1215G > A, which were identified as causative mutations of SLE in 4 of 52 Chinese pediatric patients. Three compound heterozygous mutations and one homozygous mutation in *SLC7A7* were revealed in these four patients. The mutation c.625+1G > A of *SLC7A7* was detected in 3 of 4 patients [27]. We also detected compound heterozygous variants in *SLC7A7*, namely, c.625+1G > A and c.250G > A (p. V84I), in our patient, with the former consistent with the previously reported variants. Remarkably, her father also carries a *SLC7A7* mutation, namely, c.625+1G > A, but has no history of SLE.

The GEO analysis revealed that CPOX expression was downregulated in the SLE patients compared with the normal controls (adj. *P* = 0.0071, logFC = −1.0975). The relationship between *CPOX* mutations and SLE has never been reported previously. The *CPOX* variant detected in the present patient may play a role in the coexistence of HCP and SLE.

The pathogenic genes of porphyria are clear and related to the function and activity of enzymes in the heme biosynthetic pathway. The proband was diagnosed with HCP due to a splicing mutation of *CPOX* (c.700+2 T > C). The same *SLC7A7* mutation (c.625+1G > A) was detected both in the proband and her father, although only the proband presented with SLE. We suggest that the *SLC7A7* mutation is related to the immune dysregulation of SLE and that the *CPOX* mutation promotes this process. The mentioned variants have been submitted to the ClinVar database. Further research needs to be conducted to investigate the mechanism underlying the coexistence of HCP and SLE.

## 5. Limitations

In this patient, the positive UPG and PBG and increased FEP revealed the presence of AHP, and the diagnosis of HCP was identified by a *CPOX* mutation analysis. However, neither stool metabolite testing nor CPOX enzyme activity detection was performed to further confirm HCP. In addition, we did not assess protein expression by reverse transcription assay or immunohistochemistry.

## 6. Conclusion

We reported a rare case of HCP coexisting with SLE and described the diagnosis difficulties. This is the first reported case of SLE coexisting with HCP in China. We reported a novel splicing mutation of *CPOX*, i.e., c.700+2 T > C (intron 2), as well as compound heterozygous mutations of *SLC7A7*. Urine testing of porphyrin precursors is an easy and efficient technique, while genetic testing is still the gold standard for the subtype diagnosis of porphyria. The simultaneous mutation of *CPOX* and *SLC7A7* may explain the etiopathogenetic connections of HCP and SLE. A better understanding of the pathogenesis could facilitate the prediction and prevention of acute porphyria attacks.

## Figures and Tables

**Table 1:** Main data from 15 reported cases of the coexistence of AHP and SLE.

Case	Author reference	Sex	Age	Disease sequence	Lapsed time	ANA titer	aPL	Lupus nephritis	SLE treatment	Family history AHPs	Genetic test
1	Wolfram [29]	F	30	SLE→AIP	4 months	U	U	U	NR	U	U
2	Marsden [11]	F	57	SLE→AIP	2 days	U	U	U	Chloroquine	U	U
3	Passaron et al. [12]	F	13	SLE→AIP	2 years	U	U	U	Chloroquine	U	U
4	Filiotou et al. [8]	F	31	SLE→AIP	1 year	U	U	U	Barbiturates	U	U
5	Quilichini and Guerder [30]	F	22	SLE→AIP	6 years	U	U	U	Steroids, analgesics	U	U
5	Vittori and Desaegher [31]	F	22	SLE→AIP	6 years	U	U	U	Steroids, analgesics	U	U
6	Rosemarin et al. [32]	F	39	SLE→AIP	2 years	1/640	U	Present	Steroids, phenobarbital, and propranolol	Absent	U
7	Allard and Scott [33]	F	47	SLE→AIP	15 years	NR	aCL+	Present	Steroids	Absent	U
8	Andersson and Lithner [15]	F	37	AIP→SLE	NR	NR (positive)	NR	Present	NR	NR	U
9	Alioua et al. [10]	F	24	SLE→HCP	6 months	1/80	NR	NR	Steroids	Present	U
10	Filiotou et al. [8]	F	43	AIP → SLE	20 years	1/640	NR	Absent	Thalidomide	Present	U
11	Korkmaz [9]	F	31	SLE→HCP	5 years	1/80	NR	NR	Steroids, methotrexate	U	U
12	Bharati et al. [14]	M	49	AIP→SLE	2 years	1/160	NR	NR	Steroids, MMF	Present	DNA sequencing of intron 14 of the *HMBS*: IVS14+1G→T
13	Patil et al. [13]	U	U	SLE→AIP	NR	NR	NR	NR	NR	U	U
14	Esteve-Valverde et al. [7]	M	51	SLE→AIP	10 days	1/1280	IgG-ab2GPI+	Absent	Steroids, HCQ, and azathioprine	Absent	U
15	Present case (2020)	F	30	SLE→HCP	6 years	1/640 (SLE) 1/160 (HCP)	Negative	Absent	Steroids, HCQ	Absent	WES: a splicing mutation of *CPOX*: c.700+2 T > C (intron 2)

Modified from Esteve-Valverde et al. [7]. AHPs: acute hepatic porphyrias; AIP: acute intermittent porphyria; ANA: antinuclear antibodies; SLE: systemic lupus erythematosus; HCP: hereditary coproporphyria; aPL: antiphospholipid antibodies; aCL: anticardiolipin; IgG-ab2GPI: IgG anti*β*2-GPI; MMF: mycophenolate mofetil; HCQ: hydroxychloroquine; *HMBS*: hydroxymethylbilane synthase gene; WES: whole exome sequencing; *CPOX*: coproporphyrinogen III oxidase gene; NR: not referred; U: unknown. ^a^Same patient.

## Data Availability

All data generated or analyzed during this study are included in the published article. The raw data is available upon request from the corresponding author.

## Abbreviations

ACMG: American College of Medical Genetics
AHPs: Acute hepatic porphyrias
AIP: Acute intermittent porphyria
ALADP: Aminolevulinic acid dehydratase deficient porphyria
ANA: Antinuclear antibodies
ClinVar: Clinically relevant variants
CPOX: Coproporphyrinogen III oxidase
*CPOX*: Coproporphyrinogen III oxidase gene
DEGs: Differentially expressed genes
ds-DNA: Double-stranded DNS
ESR: Erythrocyte sedimentation rate
FEP: Free erythrocyte protoporphyrin
GEO: Gene expression omnibus
HCP: Hereditary coproporphyria
HCQ: Hydroxychloroquine
HMBS: Hydroxymethylbilane synthase
*HMBS*: Hydroxymethylbilane synthase gene
ITP: Immune thrombocytopenia
MMF: Mycophenolate mofetil
PBG: Porphobilinogen
REVEL: Rare exome variant ensemble learner
SLE: Systemic lupus erythematosus
*SLC7A7*: Solute carrier family 7 member 7 gene
UPG: Uroporphyrinogen
VP: Variegate porphyria
WES: Whole exome sequences.
